# Quantum Dynamics Framework with Quantum Tunneling Effect for Numerical Optimization

**DOI:** 10.3390/e27020150

**Published:** 2025-02-01

**Authors:** Quan Tang, Peng Wang

**Affiliations:** 1 Chengdu Institute of Computer Applications, China Academy of Sciences, Chengdu 610213, China; tangquan20@mails.ucas.ac.cn; 2 School of Electronic Information, Chengdu Jincheng College, Chegndu 611731, China; 3 University of Chinese Academy of Sciences, Beijing 100049, China; 4 School of Computer and Artificial Intelligence, Southwest Minzu University, Chengdu 610225, China

**Keywords:** optimization algorithms, quantum dynamics framework, evolution process, quantum tunneling

## Abstract

In recent years, optimization algorithms have developed rapidly, especially those which introduce quantum ideas, which perform excellently. Inspired by quantum thought, this paper proposes a quantum dynamics framework (QDF) which converts optimization problems into the problem of the constrained ground state of the quantum system and analyzes optimization algorithms by simulating the dynamic evolution process of physical optimization systems in the ground state. Potential energy equivalence and Taylor expansion are performed on the objective function to obtain the basic iterative operations of optimization algorithms. Furthermore, a quantum dynamics framework based on the quantum tunneling effect (QDF-TE) is proposed which adopts dynamic multiple group collaborative sampling to improve the quantum tunneling effect of the QDF, thereby increasing the population diversity and improving algorithm performance. The probability distribution of solutions can be visually observed through the evolution of the wave function, which also indicates that the QDF-TE can strengthen the tunneling effect. The QDF-TE was evaluated on the CEC 2017 test suite and shown to be competitive with other heuristic optimization algorithms. The experimental results reveal the effectiveness of introducing a quantum mechanism to analyze and improve optimization algorithms.

## 1. Introduction

Since Holland introduced the genetic algorithm (GA) in the 1970s [1], and optimization algorithms have undergone over half a century of development, giving rise to a plethora of classic algorithms, such as the ant colony optimization (ACO) algorithm [2] and the particle swarm optimization (PSO) algorithm [3]. In recent years, an extensive array of novel algorithms, enhanced algorithms, and hybrid algorithms has been proposed. However, exploring the fundamental nature or discerning the commonalities among optimization algorithms to attain improved performance represents an urgently solvable problem at present. It is imperative to derive the core basic iterative process of optimization algorithms to construct sophisticated optimization algorithms which are apt for different application scenarios.

Some scholars have made efforts to extract the basic operation and iterative process. Examples of such research include the simplified version of PSO [4], the bare-bones differential evolution algorithm (BBDE) [5], and the bare-bones fireworks algorithm (FWA) [6]. These studies show that optimization algorithms adopt different models but still have similar basic operations, such as probability sampling, sampling step adjustment, and population strategies. Therefore, we make efforts to apply quantum dynamics theory to optimization algorithms so that similar operations which widely exist in different optimization algorithms are explained in theory, and a simple but effective basic frame of optimization algorithms is also introduced. From the perspective of quantum dynamics, the iterative process of optimization algorithms can be regarded as the evolution of a wave function with time, which is the nature of a dynamic process.

There are two reasons why we chose quantum dynamics as a theoretical basis and constructed a basic theoretical framework of optimization algorithms.

First of all, quantum theory can describe optimization problems. The quantum descriptiveness of optimization problems is proven indirectly with the emergence of quantum annealing theory. In 1994, Finnila proposed the equivalence of potential energy and believed that the ground state of the system can be obtained through quantum annealing (QA) or diffusion Monte Carlo (DMC) method [7]. In 2004, Sun J. proposed quantum particle swarm optimization (QPSO) by adopting the quantum wave function into probabilistic sampling behavior [8].

Secondly, the migration of solutions of optimization algorithms is comparable to the movement of microscopic particles. The study of quantum stochastic dynamics began with Nelson’s derivation of the Schrödinger equation through Newton’s mechanics [9]. It systematically expounded the relationship between the microscopic random force action and the macro process of system evolution [10]. Based on this theory, the evolution process from the arbitrary initial state to the final state can be deduced by analyzing microscopic forces. When using quantum dynamics to observe the iterative process of optimization algorithms, the optimization process is the evolution of a probability wave of solutions based on the time-dependent Schrödinger equation. This interpretation for optimization algorithms is essentially different from classical conditions [9].

Based on the above contents, this paper adopts the Schrödinger equation to construct a dynamic time evolutionary equation for optimization algorithms and puts forward a basic iterative structure of optimization algorithms. Without loss of generality, the one-dimensional objective function f(x) (second-order differentiability) is used as the study object in order to express the core point of this paper more clearly and concisely.

The main contributions are as follows:

1. Establish the connection between a quantum system and optimization algorithms as well as the proposed quantum dynamics framework (QDF) of optimization algorithms, which provide a profound and extensive physical explanation for the iterative evolution process of optimization algorithms.
2. The physical explanation is given for the common operations of optimization algorithms, such as random group search, optimal solutions replacing inferior solutions, and multi-scale processes.
3. Based on the QDF and quantum tunneling effect, the quantum dynamics framework with a tunneling effect (QDF-TE) is proposed. The experimental results show that this method is accurate and stable, especially for high-dimensional complex optimization problems.

The remainder of this paper is organized as follows. Section 2 introduces some related work about the application of quantum theory in optimization. Section 3 introduces the relationship between optimization problems and the Schrödinger equation, and it proposes a QDF for optimization algorithms. Section 4 analyzes the quantum tunneling effect and introduces the QDF-TE. The experimental results for the QDF-TE are shown in Section 5. Section 6 concludes this paper.

## 2. Related Work

In 1994, Finnila regarded the objective function f(x) of optimization problems as the potential energy V(x) in the quantum system, which is called potential energy equivalence (V(x)=f(x)). Therefore, the ground state of the system was regarded as the solution to optimization problems and could be obtained through the quantum annealing process or diffusion Monte Carlo (DMC) method [7]. In 1998, Japanese scientist Kadowaki proposed a solution in which the Ising model could be used in a transverse magnetic field to realize the quantum annealing process [11]. In 1999, Brooke reported in *Science* that they successfully realized a simple quantum spin model using Ising magnets in a transverse magnetic field [12].

Using a quantum mechanism in computing has been an active research topic for the last few decades. The quantum mechanism brings a new philosophy to optimization due to its underlying concepts. Based on quantum mechanisms, numerous studies have been put forward to enhance both the efficiency and accuracy of typical optimization algorithms [13,14,15].

In 1996, Narayanan and Moore used the multiverse theory to propose the quantum-inspired genetic algorithm (QIGA) to solve the traveling salesman problem [16]. In 2000, Han et al. proposed the genetic quantum algorithm (GQA) at the 2000 IEEE Conference on Evolutionary Computation, in which the concept of qubits was used to improve the randomness and the global search capability of the algorithm to solve knapsack problems [17]. In 2002, Han proposed a complete version named the quantum-inspired evolutionary algorithm (QIEA) [14]. In 2007, quantum ant colony optimization (QACO) was proposed, which also uses the qubits mechanism [18]. Such research proves that quantum theory is a powerful tool for studying optimization theory. In this kind of research, some probabilistic properties such as the state superposition mechanism and DMC method in quantum mechanics are often applied to enhance the global search ability of algorithms by improving their performance.

However, these works are far from the quantum characteristics of optimization problems themselves. A quantum mechanism can be used to explain the iterative operation process of optimization algorithms essentially and give the basic framework of optimization algorithms to provide a theoretical basis for analyzing optimization algorithms and further proposing excellent optimization algorithms.

## 3. Quantum Dynamics Framework of Optimization Algorithms

### 3.1. Schrödinger Equation in Quantum Mechanics

The time-dependent Schrödinger equation, shown in Equation (1), which is the most basic equation in quantum mechanics, is a partial differential equation describing the movement of microscopic particles. It constitutes three equivalent description methods of quantum mechanics, with the matrix mechanics proposed by Heisenberg, the path integral proposed by Feynman, and the wave function proposed by Schrödinger:

(1) $$i\hbar \frac{\partial \Psi (x,t)}{\partial t}=\left(-\frac{\hbar^{2}}{2m}\frac{\partial^{2}}{\partial x^{2}}+V\left(x\right)\right)\Psi \left(x,t\right)$$

where $\hat{H}=-\frac{\hbar^{2}}{2m}\frac{\partial^{2}}{\partial x^{2}}+V\left(x\right)$ is the Hamiltonian of the system, $-\frac{\hbar^{2}}{2m}\frac{\partial^{2}}{\partial x^{2}}$ is the kinetic energy term of the Hamiltonian, and V(x) is the potential term of the Hamiltonian. Using appropriate dimensions, and by letting $D=\frac{\hbar }{2m}$, Equation (1) can be abbreviated as follows:

(2) $$i\frac{\partial \Psi (x,t)}{\partial t}=\left(-D\frac{\partial^{2}}{\partial x^{2}}+V\left(x\right)\right)\Psi \left(x,t\right)$$

In 1927, Born put forward a probability interpretation of the wave function within the context of the Schrödinger equation [19]. He posited that the modulus square of the wave function ${\left|\Psi \left(x,t\right)\right|}^{2}$ represents the probability density of particles being present at a specific position x at time t. Moreover, he asserted that the wave function fully determines the motion state of a microscopic particle.

### 3.2. Schrödinger Equation for Optimization Problems

The one-dimensional global optimization problem is defined as follows (to simplify, only the global minimum is defined):

A global minimum $x_{0}\in X$ of one function f(x);

X→R is an input element with $f\left(x_{0}\right)\leq f\left(x\right)$∀x∈X.

According to the definition of optimization problems, from a physical point of view, when the quantum system is in the ground state, particles will appear near the lowest point of potential, well within the maximum probability. When V(x)=f(x), the probability distribution of the ground state wave function is the probability distribution of the solution for an objective function. To put forward the dynamic equation of optimization problems, two important transformations are needed:

1. The solution for the optimization problems is described by a probability distribution similar to the wave function Ψ(x,t).
2. The objective function of the optimization problems is regarded as the constrained potential energy of the quantum system, namely V(x)=f(x).

Accordingly, the dynamic equation of the optimization problems is as follows:

(3) $$i\frac{\partial \Psi (x,t)}{\partial t}=\left(-D\frac{\partial^{2}}{\partial x^{2}}+f\left(x\right)\right)\Psi \left(x,t\right)$$

To express the optimization problems more generally, Wick rotation [20] was employed for the time-dependent Schrödinger equation to transform the real time into an imaginary time τ=it. Thus, Equation (3) is rewritten as follows:

(4) $$\frac{\partial \Psi (x,\tau )}{\partial \tau }=\left(D\frac{\partial^{2}}{\partial x^{2}}-f\left(x\right)\right)\Psi \left(x,\tau \right)$$

This equation is a general form of the time-dependent Schrödinger equation of optimization problems. It is a dynamic equation which can theoretically describe evolutionary behavior for the solutions to optimization algorithms in an iterative process.

### 3.3. Taylor Approximation of Objective Function

For the objective function of the optimization problems, its analytic structure is unknown in most cases. Therefore, we can only use a sampling operation to obtain the information for the objective function.

We consider using Taylor expansion to expand the complex objective function’s neighborhood at a certain point into a simple polynomial. Taylor expansion simplifies the objective function given by Equation (5). First, the objective function f(x) is assumed to be continuously differentiable and is expanded as follows:

(5) $$f\left(x\right)=\sum_{n=0}^{\infty }\frac{f^{\left(n\right)}\left(x_{0}\right)}{n!}{\left(x-x_{0}\right)}^{n}$$

where n! represents the factorial of n and $f^{\left(n\right)}\left(x_{0}\right)$ is the nth derivative of f(x) at the point $x_{0}$. The zero-order Taylor approximation of f(x) is $f^{\left(0\right)}\left(x\right)=f\left(x_{0}\right)$, where $f(x_{0})$ is a constant. Thus, by translation, $f^{\left(0\right)}\left(x\right)=0$. The first-order Taylor approximation of f(x) is $f^{\left(1\right)}\left(x\right)=f^{\left(1\right)}\left(x_{0}\right)\left(x-x_{0}\right)$. If the point $x_{0}$ is an extreme point, then its first derivative $f^{\left(1\right)}\left(x_{0}\right)=0$, and thus the first-order Taylor approximation near the extreme point is $f^{\left(1\right)}\left(x\right)=0$. The second-order Taylor approximation is $f^{\left(2\right)}\left(x\right)=\frac{1}{2}f^{\left(2\right)}\left(x_{0}\right){\left(x-x_{0}\right)}^{2}$, assuming that the higher-order terms are negligible when $\left(x-x_{0}\right)$ is small enough.

#### 3.3.1. Dynamic Equation of Optimization Algorithms Under Zero-Order Taylor Approximation

Zero-order Taylor expansion of the objective function f(x) is approximate to a constant which does not contain guidance information. By translating the coordinate axis, the global optimal value of the function can be shifted to zero. Therefore, the objective function can be regarded as a constant equal to zero as follows:

(6) f(x)=0

The dynamic equation of the optimization problems, which is shown as Equation (4), can be recast as

(7) $$\frac{\partial \Psi (x,\tau )}{\partial \tau }=D\frac{\partial^{2}\Psi \left(x,\tau \right)}{\partial x^{2}}$$

Equation (7) is the dynamic equation obtained from the Schrödinger equation of the optimization problems under zero-order Taylor approximation. This dynamic equation does not contain any information about the objective function f(x). It represents the dynamic behavior of the random sampling search process of optimization algorithms. This equation is also the Schrödinger equation for free particles in quantum mechanics.

This equation is completely isomorphic with the ordinary diffusion equation, with a diffusion coefficient of D. Thus, the sampling process of optimization algorithms can be described and explained by the diffusion equation under quantum theory. The evolution of the iterative process of optimization algorithms is essentially a diffusion process. The diffusion process is a group behavior in physics, which shows that the sampling process of optimization algorithms should adopt the group strategy, which has been proven by the construction practice of many optimization algorithms. At present, almost all the algorithms with the best performance adopt the group strategy.

According to the dynamic equation of optimization algorithms, a sampling probe in the population (a walker) with an initial position at $x^{\prime }$ diffuses with a diffusion coefficient D. The probability of appearing at position x after Δt can be expressed by Green’s function, corresponding to the diffusion equation:

(8) $$G\left(x,x^{\prime },\Delta t\right)=\frac{1}{\sqrt{4\pi D\Delta t}}e^{-\frac{{\left(x-x^{\prime }\right)}^{2}}{4D\Delta t}}$$

By letting $\sigma =\sqrt{2D\Delta t}$, Equation (8) can be recast as

(9) $$G\left(x,x^{\prime },\Delta t\right)=\frac{1}{\sqrt{2\pi }\sigma }e^{-\frac{{\left(x-x^{\prime }\right)}^{2}}{2\sigma^{2}}}$$

where Green’s function of the diffusion equation is transformed into a normal distribution, σ is the scale parameter for the sampling, and σ is related to the diffusion coefficient D and the time interval Δt of the diffusion equation. Green’s function $G(x,x^{\prime },\Delta t)$ describes the Markov process of sampling. Let a walker’s position at time t be x(t) and the position x(t+Δt) be

(10) x(t+Δt)=x(t)+σN(0,1)

where N(0,1) is the standard normal distribution and σ is the sampling scale, which continuously decreases when the time interval Δt decreases and can gradually improve the accuracy of the search. This corresponds to the multi-scale sampling behavior, which exists in the sampling process of a large number of optimization algorithms. It can be understood that normal sampling is the best choice without any prior knowledge.

#### 3.3.2. Dynamic Equation of Optimization Algorithms Under First-Order Taylor Approximation

The first-order Taylor approximation obtains the guidance information of the objective function near $x_{0}$, and its expansion is as follows:

(11) $$f^{\left(1\right)}\left(x\right)=f^{\left(1\right)}\left(x_{0}\right)\left(x-x_{0}\right)$$

The analytic equation of f(x) is unknown, and thus we can only obtain the guidance by using the position and function value of double sampling and let $x_{0}$ be the current position and x be the new sampling position, Equation (11) is recast as

(12) $$f^{\left(1\right)}\left(x\right)\approx \frac{f\left(x\right)-f\left(x_{0}\right)}{x-x_{0}}\left(x-x_{0}\right)=f\left(x\right)-f\left(x_{0}\right)$$

Equation (12) describes the process of optimization algorithms by sampling the neighborhood near $x_{0}$ to obtain the guidance information of the objective function.

Let $\Delta f=f\left(x\right)-f\left(x_{0}\right)$, and substitute it into Equation (4) to obtain

(13) $$\frac{\partial \Psi (x,\tau )}{\partial \tau }=\left[D\frac{\partial^{2}}{\partial x^{2}}-\Delta f\right]\Psi \left(x,\tau \right)$$

Equation (13) is the dynamic equation obtained from the Schrödinger equation of the optimization problems under first-order Taylor approximation.

In order to express the influence of the guidance information of the objective function obtained in sampling process, the Fokker–Planck equation is utilized, which introduces a drift term on the basis of the diffusion equation [21] as follows:

(14) $$\frac{\partial \Psi (x,\tau )}{\partial \tau }=\left[D\frac{\partial^{2}}{\partial x^{2}}-\frac{\partial f(x)}{\partial x}\right]\Psi \left(x,\tau \right)$$

where ${\left|\Psi (x,\tau )\right|}^{2}$ is the probability distribution of the solutions at τ in the iterative process of optimization algorithms, $D\frac{\partial^{2}}{\partial^{2}x}$ is diffusion term, and $\frac{\partial f(x)}{\partial x}$ is drift term. Equation (13) and the Fokker–Planck equation are isomorphic, and thus Δf can be regarded as the drift term of the quantum dynamics equation under first-order Taylor approximation.

Equation (13) shows that there are two core driving mechanisms in the iterative process of optimization algorithms; one is the normal random sampling mechanism driven by diffusion behavior, corresponding to the diffusion term $D\frac{\partial^{2}}{\partial^{2}x}$, and the other is the guiding mechanism driven by the objective function as a conservative force field, corresponding to the drift term Δf. When Δf>0, the current potential is increasing, and the algorithm tends to prevent the walker from moving to this point. When Δf<0, the current potential is decreasing, and the algorithm tends to push the walker toward this point. The drift term Δf corresponds to the drag and guide effect of the objective function as a conservative force field in the search process, and Δf corresponds to the first-order Taylor approximation of the objective function.

#### 3.3.3. Ground-State Wave Function of Optimization Algorithms Under Second-Order Taylor Approximation

Before the Wick rotation, the second-order Taylor approximation of the objective function near the extreme point $x_{0}$ of the global optimal solution is as follows:

(15) $$f^{\left(2\right)}\left(x\right)=\frac{1}{2}f^{\left(2\right)}\left(x_{0}\right){\left(x-x_{0}\right)}^{2}$$

Equation (4) can be obtained under the second-order Taylor approximation of the objective function as follows:

(16) $$\frac{\partial \psi (x,\tau )}{\partial \tau }=\left[D\frac{\partial^{2}}{\partial x^{2}}-\frac{1}{2}f^{\left(2\right)}\left(x_{0}\right){\left(x-x_{0}\right)}^{2}\right]\psi \left(x,\tau \right)$$

Equation (16) is the Schrödinger equation of the harmonic oscillator in quantum theory. Generally, the complex vibration of the equilibrium position and Lennard-Jones potential are approximated by the harmonic oscillator potential. The ground-state wave function of a quantum system with a harmonic oscillator potential energy constraint has a normal distribution. Therefore, the normal distribution can be used as the objective criterion for the population to converge to the ground state at a certain scale in the iterative process.

### 3.4. Algorithm of Quantum Dynamics Framework

As mentioned above, zero-order Taylor approximation of an objective function is a diffusion equation, which explains the random normal sampling search behavior of optimization algorithms. The first-order Taylor approximation of an objective function can be written as a Fokker–Planck equation, which explains the mechanism of obtaining the guidance information of an objective function in the sampling process. The second-order Taylor approximation of an objective function is a quantum harmonic oscillator, which indicates that a normal distribution can be used as the ground-state convergence criterion in the iterative process of optimization algorithms.

According to the above conclusions from quantum dynamics, the algorithm process of the QDF can be given as shown in Algorithm 1.
**Algorithm 1:** General framework of QDF.
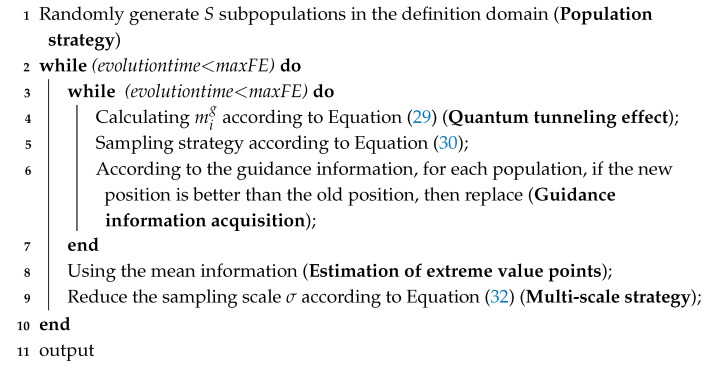


In summary, the sampling process of optimization algorithms includes three basic strategies: (1) the population strategy; (2) normal distribution sampling strategy; and (3) multi-scale strategy. These three strategies and their variants are usually used in most optimization algorithms.

In addition to the three basic strategies mentioned above, utilization of the mean information in Algorithm 1 refers to the estimation of the optimal solution region by using the current population distribution. The common methods include the introduction of an average optimal position vector in the position update equation in QPSO [22] and using the population mean coordinate to generate new individuals to replace the individuals with the worst fitness in the multi-scale quantum harmonic oscillator optimization algorithm (MQHOA). Utilization of the mean information can effectively improve the accuracy of algorithms. According to the above conclusion that the ground-state wave function of a harmonic oscillator has a normal distribution under second-order Taylor approximation of the objective function, and by taking the normal distribution as the approximate wave function of the ground state, a variance of p walkers less than σ is regarded as an approximated ground-state criterion to judge the ground state of the system.

The QDF is the core iterative process of optimization algorithms. This basic structure is highly similar to the bare-bones edition of some optimization algorithms [5,6]. An optimization algorithm with good performance could usually be formed by adding some strategies with prior knowledge of this basic iterative structure.

## 4. Quantum Dynamics Framework with the Tunneling Effect (QDF-TE)

In complex optimization problems, there are numerous local optimal regions separated by potential barriers, causing particles to become trapped and unable to find the global optimum. The quantum tunneling effect reflects that the particles in the quantum system can penetrate through the potential barrier and appear in the classically forbidden zone. In optimization problems, the particle (sampling point) has a probability of jumping from the local optimal to the global optimal region, which is called the quantum tunneling effect.

In optimization fields, the quantum tunneling effect first appeared in quantum annealing (QA) [7], which has better optimization performance than simulated annealing (SA) [23] in some cases. QA can help a system escape the local minima by using quantum tunneling through the barriers rather than thermally overcoming them, with an artificial and appropriate source of quantum fluctuations which is slowly switched off according to the annealing schedule [24]. During the annealing process, the system visits various local energy minima freely. But the annealing schedule is controlled by a specific function, such as a power law or linear function. Once the function is selected, the system can only iterate according to a fixed annealing schedule. Therefore, there is a probability that the tunneling effect cannot be controlled according to the iteration situation automatically.

Based on the QDF, this section further investigates the relationship between the probability of the tunneling effect and the fitness of the population, and it proposes the QDF-TE, which can dynamically adjust the probability of the tunneling effect based on the current fitness landscape to improve the optimization capability.

### 4.1. Motivation

According to path integration [25], the general solution of the Schrödinger equation can be written as follows:

(17) $$\psi \left(x,\tau \right)=\int_{-\infty }^{\infty }dx_{0}K\left(x,\tau |x_{0},0\right)\psi \left(x_{0},0\right)$$

where $K\left(x,\tau |x_{0},0\right)$ is the propagator, representing the probability of particles migrating from position $x_{0}$ to position *x* and from t=0 to τ as follows:

(18) $$\begin{array}{cc} \; & K\left(x,\tau |x_{0},0\right)=\lim_{N\to \infty }\int_{-\infty }^{\infty }dx_{1}\ldots \int_{-\infty }^{\infty }dx_{N-1}{\left(\frac{m}{2\pi \hbar \Delta \tau }\right)}^{\frac{N}{2}}\times \exp\left\{-\frac{\Delta \tau }{\hbar }\sum_{j=1}^{N}\left[\frac{m{\left(x_{j}-x_{j-1}\right)}^{2}}{2\Delta \tau^{2}}+V\left(x_{j}\right)-E_{R}\right]\right\} \end{array}$$

where Δτ is the time interval, and when N→∞, an accurate migration probability can be obtained.

We define the kinetic energy term $P\left(x_{n},x_{n-1}\right)$ in Equation (19), which represents the Gaussian probability density of the random variable $x_{n}$, with a mean of $x_{n-1}$ and a variance of $\sigma^{2}=\hbar \Delta \tau /m$:

(19) $$P\left(x_{n},x_{n-1}\right)={\left(\frac{m}{2\pi \hbar \Delta \tau }\right)}^{\frac{1}{2}}\exp\left[-\frac{m{\left(x_{n}-x_{n-1}\right)}^{2}}{2\hbar \Delta \tau }\right]$$

We also define the weight factor $W\left(x_{n}\right)$ in Equation (20), which is related to the potential energy and reference energy:

(20) $$W\left(x_{n}\right)=\exp\left[-\frac{\Delta \tau }{\hbar }\left(V\left(x_{n}\right)-E_{R}\right)\right]$$

We then rewrite the solution of the imaginary time Schrödinger equation as

(21) $$\begin{array}{ccc} \; & \psi \left(x,\tau \right)=\lim_{N\to \infty }\int_{-\infty }^{\infty }\left(\prod_{j=0}^{N-1}dx_{j}\right)\times & \prod_{n=1}^{N}W\left(x_{n}\right)P\left(x_{n},x_{n-1}\right)\psi \left(x_{0},0\right) \end{array}$$

This formula can only be solved for a specific V(x) value. When the objective function is unknown for some optimization problems, the Monte Carlo method is used to solve the formula. The basic idea is to consider the wave function itself a probability density.

The wave function modulus represents the probability distribution of particles in the feasible solution space. The wave function of the system at a certain moment τ is approximated as the superposition of all particle sampling functions. According to Equation (10), new solutions are obtained through normal sampling. We can calculate the probability distribution of a single particle using the following equation:

(22) $$\psi \left(x\right)=\int_{-\infty }^{\infty }\frac{1}{\sqrt{2\pi }\sigma }e^{-\frac{{\left(x-x_{1}\right)}^{2}}{2\sigma^{2}}}\;dx$$

where $x_{1}$ is the position of the first particle and $x_{1}$ is also the mean. Therefore, the probability distribution of *k* particles can be calculated using Equation (23):

(23) $$\psi_{s}\left(x\right)=\sum_{i=1}^{k}\int_{-\infty }^{\infty }\frac{1}{\sqrt{2\pi }\sigma }e^{-\frac{{\left(x-x_{i}\right)}^{2}}{2\sigma^{2}}}\;dx$$

where $\psi_{s}\left(x\right)$ is the system wave function, $x_{i}$ is the position of the ith particle, and $x_{i}$ is also the mean. Correspondingly, the tunneling effect probability of a particle can be approximately calculated using the normal distribution probability density as follows:

(24) $$P_{te}\left(x\right)=\int_{x_{1}-\Delta }^{x_{1}+\Delta }\frac{1}{\sqrt{2\pi }\sigma }e^{-\frac{{\left(x-x_{1}\right)}^{2}}{2\sigma^{2}}}\;dx$$

where $P_{te}\left(x\right)$ is the probability of the tunneling effect of a particle at time τ in $x_{1}\pm \Delta$ on the horizontal axis and Δ is positively correlated with the sampling step size σ.

At the beginning iteration, the sampling step size σ is relatively large, and $P_{te}\left(x\right)$ is also correspondingly large, which can meet the optimization requirements. But when σ is small in the later stage of iteration, $P_{te}\left(x\right)$ is small, and the tunneling effect is weakened. It is difficult to escape from local optima for some optimization problems with small local optima structures.

According to Equation (24), the tunneling ability of a single particle is defined by the sampling scale, and increasing the number of sampling particles *k* can improve the tunneling ability of the system. But if we only increase *k*, the performance of the algorithm will decline because it affects the sampling rate of the algorithm. The sampling rate refers to the proportion of the number of sampling points to the solution space, which is defined as

(25) $$\eta =\frac{k\times n}{(d_{\max}-d_{\min})/\varepsilon }$$

where *n* is the total number of iterations for function testing, $d_{\max}$ and $d_{\min}$ are the upper lower bounds of the domain, respectively, and ε is the calculation accuracy. Smaller η values can achieve higher efficiency. Merely increasing the total number of particles *k* to improve the tunneling ability of the system is not feasible. Using multi-population imbalanced sampling methods in the QDF-TE to increase tunneling effects can address this issue.

### 4.2. Proposal of QDF-TE

In the QDF-TE, the *k* sampling points are divided into *S* subpopulations. Then, the size of each subpopulation $X_{i}\left(i=1,2,3\ldots S\right)$ is adjusted according to the fitness difference of the subpopulations, where poor fitness will gain more particles. We use the dynamic tunneling effect to allocate the number of subpopulation particles for non-equilibrium sampling. The expansion of the poor subpopulation size increases the probability of the population reaching the optimal global region through quantum tunneling, and thus the migration ability of the subpopulation to the optimal region is improved. On the premise of ensuring search efficiency, this strategy aims to concentrate more computing resources on the solution space with poor fitness of subpopulations to improve the global search capability.

Indeed, this strategy has significant advantages from the perspective of exploration ability. On the one hand, these additional particles can increase the coverage of the search space. In complex optimization problems, the global optimal solution may be hidden in rather remote or difficult-to-reach areas, while populations with poor fitness may be in “barren zones” in the search space. By allocating more particles, the search range can be wider, and new potential areas are more likely to be discovered. On the other hand, from an evolutionary perspective, although these additional particles may not have shown significant advantages at present, they provide a guarantee for the diversity of the population. In the subsequent iteration process, as the environment and search state change, these seemingly useless particles may become key factors in finding the global optimal solution due to changes in certain conditions.

However, this strategy inevitably brings about a significant increase in computational costs, and many of these additional particles assigned to populations with poor fitness may not produce any useful results in the actual search process. A large amount of meaningless particle computing significantly prolongs the running time of the entire algorithm, especially for scenarios which have limitations on their computing times and resources.

In the QDF-TE, the total number of particles is fixed, and the computational cost will be relatively lower compared with the case where the total number of particles increases. At the same time, in the later stage of optimization, as the sampling scale decreases, particles gradually gather, and the distance between subpopulations gradually approaches zero. The size difference between subpopulations decreases, and the advantage of subpopulations with poor fitness obtaining more particles also decreases. Therefore, in the QDF-TE, the algorithm mainly consumes resources during early exploration to find the global optimum. In the future, we will further explore the computational resource cost problem of optimization algorithms.

Based on this situation, we need further weigh and choose the optimization algorithm in the real world [26]. If there is a scenario with strict requirements for the global optimal solution or relatively sufficient computing resources, then this strategy is a better choice.

The QDF-TE mainly consists of three steps: acquisition of reference energy (ER), determination of the subpopulation size, and Monte Carlo sampling.

#### 4.2.1. Acquisition of Reference Energy $E_{R}$

The fitness value of the optimal solution in the current iterative population is taken as the reference energy of the system, as shown in the following equation:

(26) $$E_{R}^{g}=f\left(x_{b}^{g}\right)=min\left(f\left(x_{i}^{g}\right)\right)$$

where $E_{R}^{g}$ is the system reference energy for the *g*th iteration, $f(x_{i}^{g})$ is the fitness value of the *i*th particle in the *g*th iteration, and $x_{b}^{g}$ is the reference solution. A potential barrier is formed from the optimal value of each subpopulation to the reference energy $E_{R}$, and the particles of each subpopulation are redistributed through the tunneling effect. The subpopulation with a larger distance to the reference solution $x_{b}^{g}$ (i.e., the subpopulation with poorer fitness values) will obtain more sampling particles. By using the tunneling effect, more sampling particles will appear in poor areas, which increases the global search capability of the algorithm.

#### 4.2.2. Determination of Subpopulation Size

Based on the distance between the optimal solution $x_{i\_b}^{g}$ of the subpopulation and the reference solution $x_{b}^{g}$, as shown in Equation (27), the size of the subpopulation is determined:

(27) $$d\left(x_{i_{-}b}^{g},x_{b}^{g}\right)=∥x_{i_{-}b}^{g},x_{b}^{g}∥$$

where $x_{i\_b}^{g}$ represents the optimal solution of the *i*th subpopulation in the *g*th iteration. The calculation of the weight factors $W(x_{n})$ in Equation (20) can lead to dynamic changes in the total number of particles in the population, which may result in the algorithm not converging or converging quickly. Since the total number of particles *k* in this algorithm is fixed, adjusting the weight factor $W(x_{n})$ of the subpopulation to change the number of particles obtained by the subpopulation will not cause the convergence problem mentioned above. Adjusting the weight factor $W(x_{n})$ through Equation (28) to satisfy $\sum_{i=1}^{S}W\left(x_{i\_b}^{g}\right)=1$. W(x) is the key to controlling the quantum tunneling of subpopulations:

(28) $$W\left(x_{i_{-}b}^{g}\right)=\frac{d\left(x_{i_{-}b}^{g},x_{b}^{g}\right)}{\sum_{i=1}^{S}\;d\left(x_{i_{-}b}^{g},x_{b}^{g}\right)}$$

Correspondingly, the number of particles obtained by each subpopulation is

(29) $$m_{i}^{g}=W\left(x_{i_{-}b}^{g}\right)\cdot k$$

where $m_{i}^{g}$ is the number of particles in the *i*th subpopulation of the *g*th iteration, which is the size of the *i*th subpopulation.

#### 4.2.3. Monte Carlo Sampling

During the migration process of particles, Monte Carlo sampling is used to determine the positions of new solutions. Equation (19) is the Monte Carlo sampling function.

For each subpopulation, their regeneration is as follows:

(30) $$X_{i}^{g+1}=N\left(X_{i}^{g},{\mathrm{cov}}_{g}\right)$$

(31) $${\mathrm{cov}}_{g}=\left(\begin{array}{ccc} \sigma_{g}^{2} & \; & \;\\ \; & \ldots & \;\\ \; & \; & \sigma_{g}^{2} \end{array}\right)$$

(32) $$\sigma_{g+1}=\sigma_{g}/C_{r}$$

where $X_{i}^{g}$ is the *i*th population of the *g*th iteration, $\sigma_{g}$ is the scale of the *g*th iteration, and $C_{r}$ is a scale reduction coefficient, usually taken to be $C_{r}=1.2$.

### 4.3. Algorithm of QDF-TE

Algorithm 2 shows the complete process of the QDF-TE. Compared with the QDF, the QDF-TE adds Equations (29) and (30). Equation (29) dynamically adjusts the size of the subpopulation based on the fitness values and increases sampling in areas with poor fitness to improve particle tunneling effects, correspondingly increasing the population diversity. Equation (30) adopts the same scale for normal sampling of each particle in the subpopulation.
**Algorithm 2:** General framework of QDF-TE.
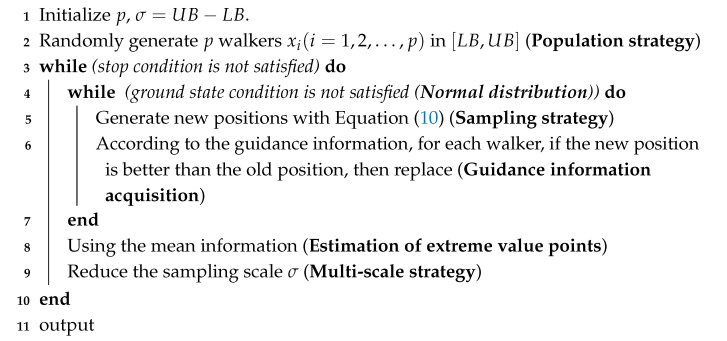



## 5. Experimental Results and Discussion

### 5.1. Experiment for Quantum Tunneling Effect

The dynamic evolution of the wave function over time actually reflects the process of the sampling population of optimization algorithms migrating to the optimal solution region. The wave function represents the probability distribution of the solutions (sampling particles). To explore the quantum tunneling effect of the QDF-TE, a wave function tracking experiment was designed according to Algorithm 2 (QDF-TE).

We selected a double-well function (DWF) as the objective function [27]. The formula of the DWF is as follows:

(33) $$f\left(x\right)=\sum_{i=1}^{n}(V_{0}\frac{{\left(x_{i}^{2}-a^{2}\right)}^{2}}{a^{4}}+\delta x_{i})$$

where i∈N , *N* is the dimension of the DWF, and the extreme value of f(x) is obtained near x=±a. When δ>0, the global minimum −aδ is obtained near x=−a, and when δ=0, the DWF has $2^{N}$ symmetric global optimal solutions. The height difference between the minima can be adjusted by changing the value of δ. $V_{0}$ is the barrier height of the DWF, which is obtained at x=0.

**Figure 1:**
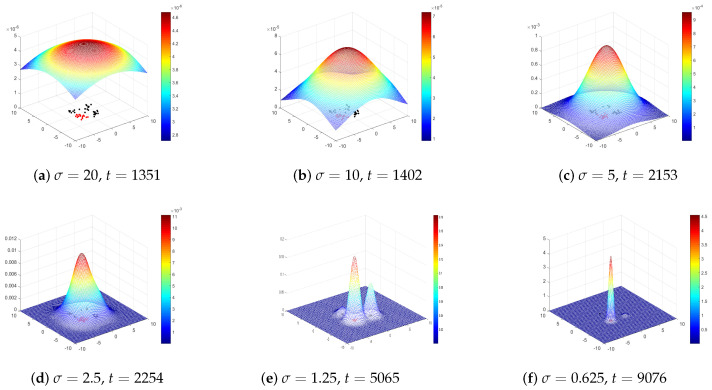
Two-dimensional schematic diagram of wave function changing with time when $V_{0}=3.0$, a=2.0, and δ=0.3.

In the experiment, a two-dimensional DWF was adopted which had four extreme points (one global optimum and two local optima). The function definition field [LB,UB] was set to [−10,10], the initial sampling scale σ=20, the sampling points k=400, and the number of subpopulations S=30. The evolution process of the wave function with time could be mapped to the reduction process of the algorithm sampling scale σ, where the reduction coefficient of σ was set to 1.2. The evolution process of the wave function over time is shown in Figure 1.

In Figure 1, *t* is the number of iterations, and subgraphs (a–f) show the evolution process of the wave function from a high-energy state to the ground state, respectively. The particles which entered the global optimum are represented in red, while the others are black. In Figure 1a, the initial image of the wave function represents the random distribution of particles throughout the entire domain. Figure 1b–d shows particles crossing the potential barrier of the objective function and continuously approaching four extreme points. Figure 1e contains three peaks, corresponding to two local optima and one global optimum, with significantly more particles clustered in the global optimum than in the local optima. As shown in Figure 1f, the wave function has a steep peak, indicating that the vast majority of the particles entered the global optimal region through the tunneling effect, and the system eventually evolved to the ground state.

### 5.2. Comparison with Other Typical Evolutionary Algorithms

#### 5.2.1. Experimental Settings

In this subsection, the QDF-TE is compared with some classic and competitive swarm intelligent optimization algorithms, including ABC [28], dynamic search FWA (dynFWA) [29], loser-out tournament-based FWA (LOTFWA) [30], PVADE [31], and standard PSO 2011 (SPSO2011) [32]. The parameters of these algorithms were set to the suggested values in their original papers. The parameter settings of the QDF-TE were consistent with Section 5.1. All of these algorithms were tested under the same conditions in the CEC 2017 single-objective optimization benchmark suite [33], which includes unimodal functions (F1–F3), multimodal functions (F4–F10), hybrid functions (F11–F20), and composition functions (F21–F30).

Experiments were conducted on 30D and 50D problems for each test function. The results of each experiment were obtained based on 51 independent trial runs, and the maximum number of function evaluations (MaxFES) in each run was 10,000×N, where *N* is the dimension of the problem. When the difference between the best solution found and the best solution was $10^{-8}$ or less, the error was zero.

#### 5.2.2. Comparative Experiments

The statistical results are listed in Table 1 and Table 2, where the best values for each function are highlighted. The average ranking (AR) of the mean errors (MeanErr) and the standard deviations (StdErr) of the six algorithms were calculated separately for the unimodal, multimodal, hybrid, and composition functions as well as all functions. The MeanErr values were adopted for comparison using the Wilcoxon signed-rank test (confidence level of 95%), where the symbols “+”, “≈”, and “−” indicate that the proposed algorithm performed better than, comparable to, or worse than the compared algorithm, respectively.

For unimodal functions, SPSO2011 showed excellent performance and ranked first for the 30D and 50D problems. DynFWA, LOTFWA, and PVADE were tied for second place for the 30D problems. The QDF-TE and ABC were ranked in fourth and fifth place, respectively. However, for the 50D problems, the QDF-TE ranked second, and for the StdErr, the QDF-TE ranked first, showing the best stability.

In terms of multimodal functions, the QDF-TE ranked first in terms of absolute advantage, especially on F5, F7, and F8 for both the 30D and 50D problems. LOTFWA and ABC achieved comparable performances, ranking second and third, respectively. DynFWA was ranked last.

For hybrid functions, PVADE was obviously superior and far ahead of the other algorithms. Although not as good as PVADE, the QDF-TE performed better than the other four algorithms. Compared with the 30D problems, the AR of the QDF-TE significantly improved for the 50D problems.

With respect to the composition functions, ABC and the QDF-TE had comparable performances for the 30D problems, while for the 50D problems, the QDF-TE was slightly more accurate than ABC. In terms of the StdErr, the QDF-TE definitively ranked first for both 30D and 50D problems, indicating that the algorithm had improved stability.

Based on the above results in the two tables, the QDF-TE was the most competitive framework among the above-mentioned algorithms for solving the multimodal and composition optimization problems, especially for high-dimensional functions. For hybrid functions, the QDF-TE performed slightly worse but was also competitive. Overall, due to the quantum tunneling effect of the QDF-TE, which can cross potential barriers and guide particles from local optima to global optima, it performed well for high-dimensional objective functions with a massive number of local optima. However, for unimodal functions, the tunneling ability of the QDF-TE had no significant effect, and thus its performance had no significant improvement.

The last row of Table 1 and Table 2 provides the overall results for the Wilcoxon signed-rank test, from which it can be seen that the performance of the QDF-TE was slightly worse than PVADE in the 30D problems, while in the 50D problems, the QDF-TE obviously outperformed all five other algorithms; that is to say, the QDF-TE performed excellently in high-dimensional function optimization.

#### 5.2.3. Convergence Discussion of QDF-TE

**Figure 2:**
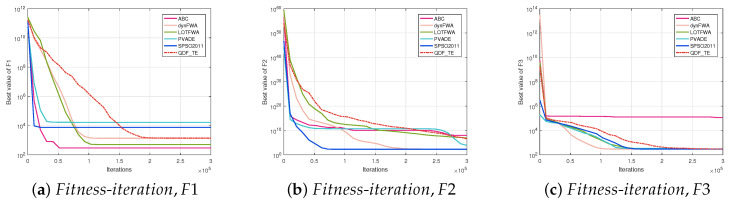

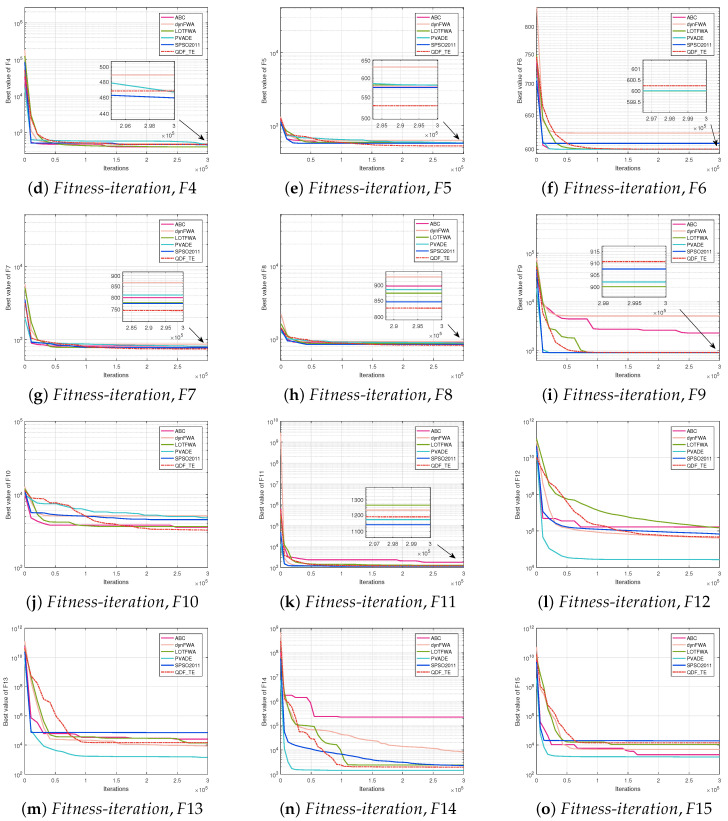
Fitness-iteration comparison among ABC, dynFWA, SPSO2011, GWO, and QDF-TE for 30 dimensional function evaluations (F1–F15).

**Figure 3:**
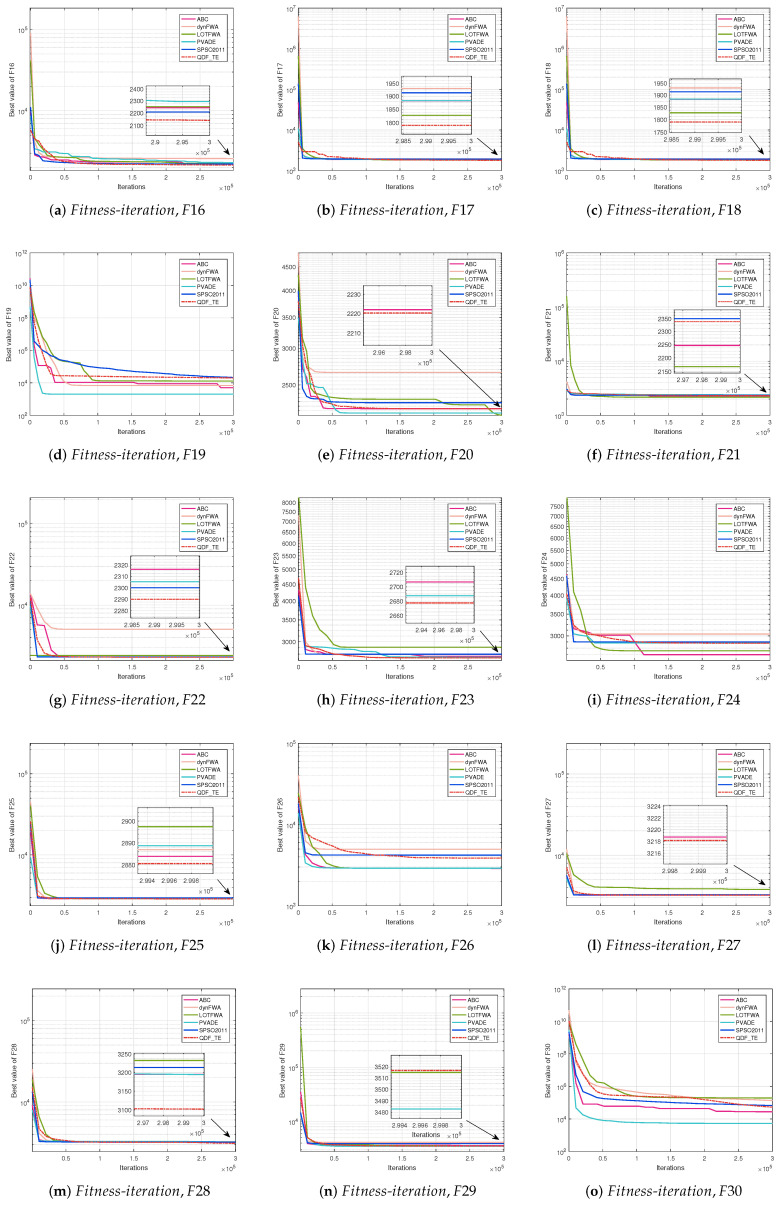
Fitness-iteration comparison among ABC, dynFWA, SPSO2011, GWO, and QDF-TE for 30 dimensional function evaluations (F16–F30).

**Figure 4:**
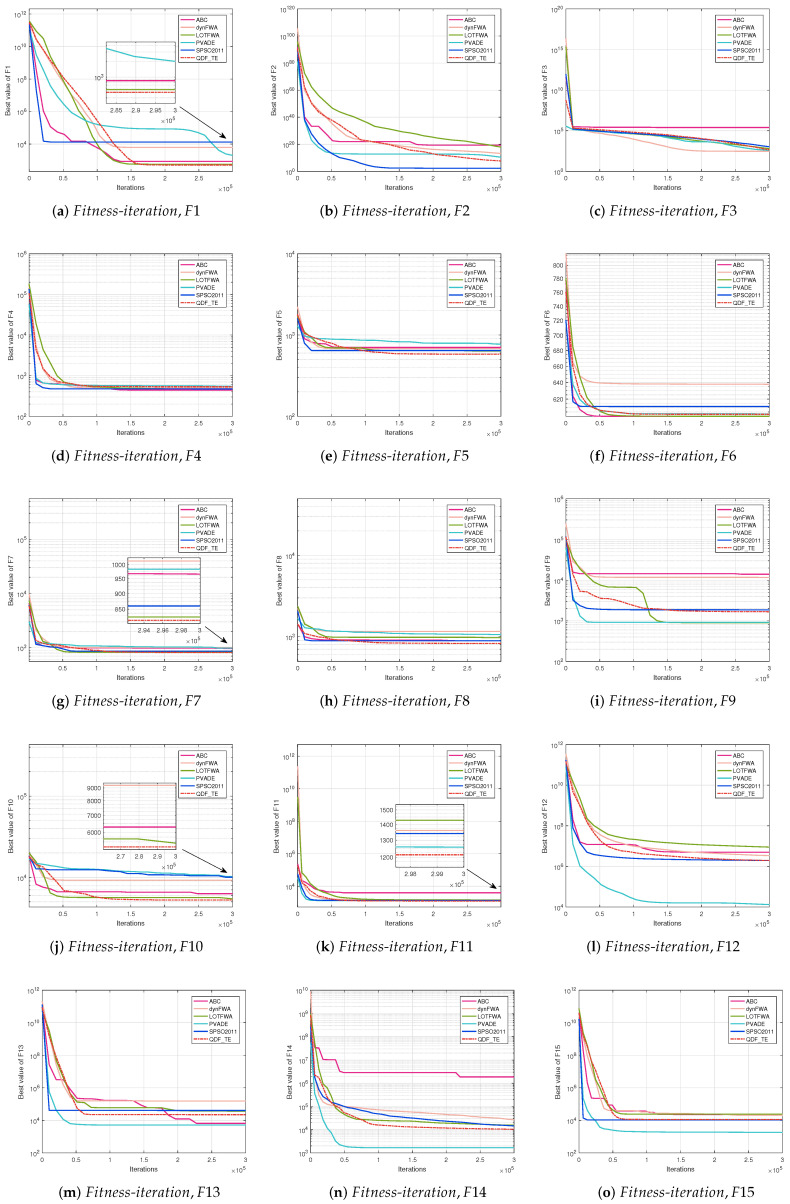
Fitness-iteration comparison among ABC, dynFWA, SPSO2011, GWO, and QDF-TE for 50 dimensional function evaluations (F1–F15).

**Figure 5:**
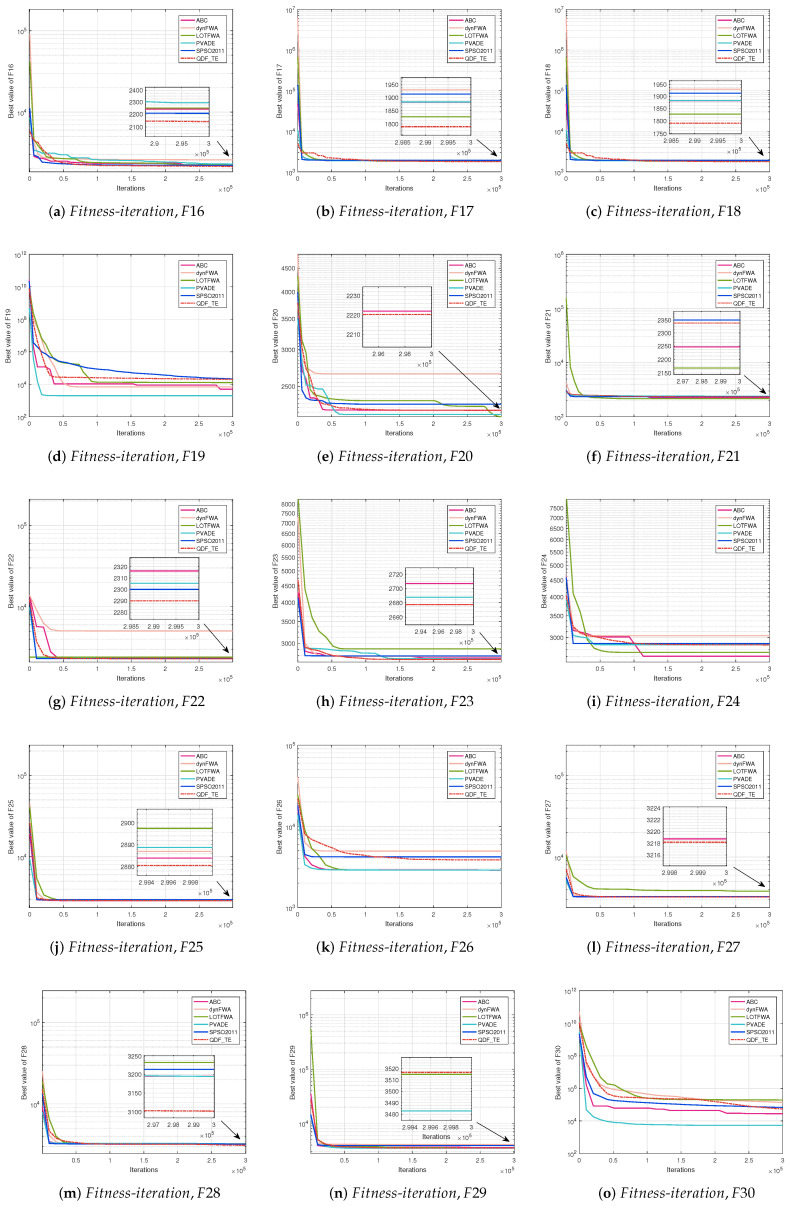
Fitness-iteration comparison among ABC, dynFWA, SPSO2011, GWO, and QDF-TE for 50 dimensional function evaluations (F16–F30).

The convergence curve is a relation between the values of fitness functions and the number of iterations for all competitive algorithms. The convergence curve plots the best fitness value at a certain number of iterations until the maximum number of iterations of the algorithm is met. Figure 2 (30D, F1–F15), Figure 3 (30D, F16–F30), Figure 4 (50D, F1–F15), and Figure 5 (50D, F16–F30) display the convergence curves of ABC, dynFWA, SPSO2011, GWO, and the QDF-TE on the CEC2017 benchmark suite, respectively.

In Figure 2 and Figure 3, for the 30D problems, the QDF-TE was able to find the optimal solution with high accuracy among 12 functions, including F5, F7, F8, F10, F16, F17, F18, F22, F23, F25, F27, and F28, showing that the QDF-TE ranked first among all six algorithms, and the overall performance of the QDF-TE was powerful. Due to the application of the tunneling effect to enhance the global search capability, the convergence speed of the QDF-TE was not the fastest at the beginning of iteration. However, once it found the global optimal region, the later exploration process would be fast.

Meanwhile, in Figure 4 and Figure 5, for the 50D problems, the QDF-TE was able to find the optimal solution with high accuracy among 15 functions, adding 3 functions (F1, F10, and F24) compared with the 30D problems. The QDF-TE still ranked first among all six algorithms. As composition functions have a large number of local optima, especially when the dimension increases, the local optimum increases exponentially, which requires an algorithm with excellent performance. Due to the dynamic population and tunneling effect mechanism, the QDF-TE could explore the solution space more thoroughly. Therefore, the QDF-TE had excellent performance in optimizing the combination functions. From Figure 5, it can be seen that the QDF-TE ranked first in the composite functions, including F22, F23, F24, F25, F27, and F28, which shows that the overall performance of the QDF-TE was so powerful that it could perform a smoother transition between exploration and exploration trends.

## 6. Conclusions

This paper introduced the Schrödinger equation to obtain a quantum dynamics equation, The QDF was further constructed using potential energy equivalence and Taylor expansion. The basic operations of optimization algorithms were obtained. A wave function was introduced to simulate the distribution of solutions in optimization algorithms. Furthermore, the QDF-TE was proposed, which divides subpopulations based on fitness values and increases the sampling capability for disadvantaged areas to strengthen the tunneling effect. Theoretical analysis and experimental results showed that this method is accurate and stable. Moreover, the results of the tunneling experiment and a comparison with other well-known optimization algorithms showed that the QDF-TE has comparable performance for complex optimization problems. However, as mentioned in Section 4.2, further research may be needed in the future, such as how to improve the utilization efficiency of these additional particles and reduce unnecessary computational overhead while ensuring the exploration capability.

The QDF-TE algorithm exhibited considerable performance. This outcome suggests that the incorporation of quantum dynamics for the purpose of explaining and enhancing optimization algorithms holds great promise. However, there remains substantial room for further improvement of the QDF algorithm. For instance, the utilization of adaptability scales for sampling across different dimensions and within subpopulations could be employed to precisely realize tunneling effects. Consequently, the potential of quantum dynamics can be explored in greater depth. Undoubtedly, this exploration represents an intriguing and potentially fruitful area of research which merits further investigation in the future.

## Figures and Tables

**Table 1:** Comparison of statistical results evaluated by five algorithms on 30D CEC 2017 benchmark functions.

F	ABC			dynFWA			LOTFWA			PVADE			SPSO2011			QDF-TE	
	**MeanErr**	**Win**	**StdErr**	**MeanErr**	**Win**	**StdErr**	**MeanErr**	**Win**	**StdErr**	**MeanErr**	**Win**	**StdErr**	**MeanErr**	**Win**	**StdErr**	**MeanErr**	**StdErr**
1	$2.28\times 10^{2}$	+	$6.13\times 10^{4}$	$4.12\times 10^{3}$	+	$1.99\times 10^{7}$	$1.17\times 10^{2}$	−	$3.36\times 10^{4}$	$3.15\times 10^{3}$	≈	$1.35\times 10^{7}$	$3.36\times 10^{3}$	+	$9.89\times 10^{6}$	$1.70\times 10^{3}$	$1.51\times 10^{6}$
2	$5.82\times 10^{9}$	−	$2.67\times 10^{20}$	$7.38\times 10^{1}$	−	$3.43\times 10^{4}$	$4.81\times 10^{9}$	−	$1.47\times 10^{20}$	$2.36\times 10^{4}$	−	$4.47\times 10^{9}$	$6.29\times 10^{-5}$	−	$1.39\times 10^{-9}$	$5.11\times 10^{8}$	$7.91\times 10^{17}$
3	$1.11\times 10^{5}$	−	$2.55\times 10^{8}$	$6.16\times 10^{-12}$	−	$1.74\times 10^{-22}$	2.87	−	7.47	$5.74\times 10^{-6}$	−	$7.97\times 10^{-11}$	0.00	−	0.00	6.65	$1.48\times 10^{1}$
AR.1-3	4.67		4.67	3.33		3.33	3.33		3.33	3.33		3.67	**2.33**		**2.00**	4.00	4.00
4	$1.29\times 10^{1}$	+	$2.97\times 10^{2}$	$7.97\times 10^{1}$	+	$8.31\times 10^{2}$	$6.51\times 10^{1}$	≈	$1.22\times 10^{3}$	$5.75\times 10^{1}$	≈	$9.69\times 10^{2}$	$5.85\times 10^{1}$	≈	$2.11\times 10^{3}$	$6.79\times 10^{1}$	$1.32\times 10^{3}$
5	$8.51\times 10^{1}$	−	$1.53\times 10^{2}$	$1.28\times 10^{2}$	+	$2.02\times 10^{3}$	$6.46\times 10^{1}$	−	$1.13\times 10^{2}$	$7.91\times 10^{1}$	−	$2.09\times 10^{2}$	$6.03\times 10^{1}$	−	$1.68\times 10^{2}$	$2.80\times 10^{1}$	$3.71\times 10^{1}$
6	0.00	+	0.00	$2.37\times 10^{1}$	+	$1.02\times 10^{2}$	$1.01\times 10^{-1}$	+	$6.89\times 10^{-2}$	$1.89\times 10^{-1}$	+	$1.28\times 10^{-1}$	8.66	+	$1.33\times 10^{1}$	$8.77\times 10^{-1}$	$1.73\times 10^{-1}$
7	$1.07\times 10^{2}$	−	$7.79\times 10^{1}$	$2.00\times 10^{2}$	+	$2.17\times 10^{3}$	$6.42\times 10^{1}$	−	$5.40\times 10^{1}$	$1.13\times 10^{2}$	+	$8.67\times 10^{1}$	$8.73\times 10^{1}$	−	$2.24\times 10^{2}$	$5.00\times 10^{1}$	$2.00\times 10^{1}$
8	$9.16\times 10^{1}$	−	$1.72\times 10^{2}$	$1.31\times 10^{2}$	+	$1.14\times 10^{3}$	$6.80\times 10^{1}$	−	$2.13\times 10^{2}$	$7.64\times 10^{1}$	−	$9.80\times 10^{1}$	$5.18\times 10^{1}$	−	$1.51\times 10^{2}$	$2.22\times 10^{1}$	$2.06\times 10^{1}$
9	$1.19\times 10^{3}$	−	$2.32\times 10^{5}$	$3.57\times 10^{3}$	+	$2.78\times 10^{6}$	$2.89\times 10^{1}$	−	$3.29\times 10^{4}$	5.68	≈	$2.43\times 10^{1}$	$1.16\times 10^{2}$	−	$1.33\times 10^{4}$	8.02	$6.44\times 10^{1}$
10	$2.47\times 10^{3}$	≈	$4.96\times 10^{4}$	$3.92\times 10^{3}$	+	$3.50\times 10^{5}$	$2.35\times 10^{3}$	≈	$1.44\times 10^{5}$	$4.06\times 10^{3}$	+	$1.37\times 10^{5}$	$3.93\times 10^{3}$	+	$5.47\times 10^{5}$	$2.48\times 10^{3}$	$7.38\times 10^{4}$
AR.4-10	3.29		2.57	5.71		5.29	2.57		3.29	3.57		3.00	3.43		4.57	**2.43**	**2.29**
11	$7.90\times 10^{2}$	−	$2.50\times 10^{5}$	$1.24\times 10^{2}$	−	$1.94\times 10^{3}$	$1.51\times 10^{2}$	−	$1.46\times 10^{3}$	$6.09\times 10^{1}$	−	$1.17\times 10^{3}$	$1.13\times 10^{2}$	−	$1.31\times 10^{3}$	$7.13\times 10^{1}$	$3.08\times 10^{2}$
12	$1.01\times 10^{6}$	−	$1.49\times 10^{11}$	$9.13\times 10^{5}$	−	$8.57\times 10^{11}$	$8.35\times 10^{6}$	+	$3.06\times 10^{13}$	$8.38\times 10^{3}$	−	$4.97\times 10^{7}$	$4.01\times 10^{5}$	≈	$8.11\times 10^{10}$	$4.16\times 10^{5}$	$7.37\times 10^{10}$
13	$1.56\times 10^{4}$	+	$6.95\times 10^{7}$	$2.35\times 10^{4}$	≈	$2.71\times 10^{8}$	$1.52\times 10^{4}$	−	$4.56\times 10^{7}$	$2.97\times 10^{2}$	−	$2.09\times 10^{5}$	$5.79\times 10^{4}$	+	$9.56\times 10^{8}$	$2.32\times 10^{4}$	$3.12\times 10^{7}$
14	$1.61\times 10^{5}$	−	$1.02\times 10^{10}$	$5.15\times 10^{3}$	−	$1.21\times 10^{7}$	$2.28\times 10^{3}$	−	$4.11\times 10^{6}$	$5.78\times 10^{1}$	−	$3.06\times 10^{2}$	$1.68\times 10^{3}$	−	$4.16\times 10^{6}$	$5.68\times 10^{2}$	$1.23\times 10^{5}$
15	$3.30\times 10^{3}$	+	$7.06\times 10^{6}$	$1.26\times 10^{4}$	≈	$1.38\times 10^{8}$	$7.61\times 10^{3}$	+	$1.32\times 10^{7}$	$9.57\times 10^{1}$	−	$3.84\times 10^{3}$	$2.92\times 10^{4}$	+	$2.28\times 10^{8}$	$1.11\times 10^{4}$	$1.30\times 10^{7}$
16	$6.36\times 10^{2}$	+	$1.32\times 10^{4}$	$9.78\times 10^{2}$	+	$8.68\times 10^{4}$	$4.95\times 10^{2}$	≈	$1.52\times 10^{4}$	$4.94\times 10^{2}$	≈	$7.34\times 10^{4}$	$7.19\times 10^{2}$	+	$1.01\times 10^{5}$	$4.87\times 10^{2}$	$1.34\times 10^{4}$
17	$1.99\times 10^{2}$	−	$2.81\times 10^{3}$	$4.08\times 10^{2}$	+	$3.26\times 10^{4}$	$1.68\times 10^{2}$	−	$3.36\times 10^{3}$	$1.06\times 10^{2}$	≈	$3.62\times 10^{3}$	$1.85\times 10^{2}$	−	$6.41\times 10^{3}$	$1.05\times 10^{2}$	$1.26\times 10^{3}$
18	$3.09\times 10^{5}$	−	$2.15\times 10^{10}$	$1.48\times 10^{5}$	−	$8.93\times 10^{9}$	$4.15\times 10^{4}$	−	$4.29\times 10^{8}$	$1.72\times 10^{2}$	−	$4.49\times 10^{3}$	$6.41\times 10^{4}$	−	$9.65\times 10^{8}$	$4.90\times 10^{4}$	$4.35\times 10^{8}$
19	$9.36\times 10^{3}$	+	$3.63\times 10^{7}$	$1.64\times 10^{4}$	+	$2.45\times 10^{8}$	$1.32\times 10^{4}$	+	$1.31\times 10^{8}$	$5.21\times 10^{1}$	−	$1.02\times 10^{3}$	$2.35\times 10^{4}$	+	$9.76\times 10^{7}$	$5.04\times 10^{4}$	$5.55\times 10^{8}$
20	$2.68\times 10^{2}$	≈	$4.50\times 10^{3}$	$4.51\times 10^{2}$	+	$3.75\times 10^{4}$	$2.80\times 10^{2}$	≈	$4.48\times 10^{3}$	$1.46\times 10^{2}$	−	$1.68\times 10^{3}$	$2.74\times 10^{2}$	≈	$6.69\times 10^{3}$	$2.67\times 10^{2}$	$3.64\times 10^{3}$
AR.11-20	4.20		3.70	5.00		5.20	3.60		3.50	**1.20**		**1.70**	4.20		4.50	2.80	2.40
21	$2.19\times 10^{2}$	≈	$6.18\times 10^{3}$	$3.19\times 10^{2}$	+	$9.59\times 10^{2}$	$1.00\times 10^{2}$	−	$4.38\times 10^{-3}$	$2.84\times 10^{2}$	+	$1.96\times 10^{2}$	$2.52\times 10^{2}$	+	$2.23\times 10^{2}$	$2.27\times 10^{2}$	$6.48\times 10^{1}$
22	$1.13\times 10^{2}$	−	$7.53\times 10^{1}$	$2.38\times 10^{3}$	+	$3.68\times 10^{6}$	$1.00\times 10^{2}$	−	$1.65\times 10^{-2}$	$1.02\times 10^{2}$	≈	5.62	$3.12\times 10^{2}$	+	$7.45\times 10^{5}$	$1.00\times 10^{2}$	$5.18\times 10^{-8}$
23	$4.22\times 10^{2}$	−	$4.04\times 10^{2}$	$5.02\times 10^{2}$	+	$1.49\times 10^{3}$	$6.16\times 10^{2}$	+	$1.49\times 10^{3}$	$3.96\times 10^{2}$	−	$6.01\times 10^{2}$	$4.25\times 10^{2}$	+	$4.68\times 10^{2}$	$3.78\times 10^{2}$	$6.39\times 10^{1}$
24	$3.43\times 10^{2}$	+	$3.06\times 10^{4}$	$5.57\times 10^{2}$	+	$1.80\times 10^{3}$	$2.92\times 10^{2}$	−	$7.37\times 10^{2}$	$4.55\times 10^{2}$	+	$2.04\times 10^{2}$	$4.83\times 10^{2}$	+	$3.76\times 10^{2}$	$4.41\times 10^{2}$	$4.06\times 10^{1}$
25	$3.84\times 10^{2}$	+	$8.33\times 10^{-2}$	$3.89\times 10^{2}$	+	$8.72\times 10^{1}$	$4.10\times 10^{2}$	+	$2.15\times 10^{2}$	$4.03\times 10^{2}$	+	$3.67\times 10^{2}$	$4.18\times 10^{2}$	+	$6.40\times 10^{2}$	$3.81\times 10^{2}$	2.53
26	$3.13\times 10^{2}$	+	$4.59\times 10^{3}$	$2.60\times 10^{3}$	+	$4.47\times 10^{5}$	$4.37\times 10^{2}$	+	$3.11\times 10^{5}$	$4.65\times 10^{2}$	+	$1.91\times 10^{5}$	$1.53\times 10^{3}$	+	$5.56\times 10^{5}$	$1.07\times 10^{3}$	$1.81\times 10^{5}$
27	$5.12\times 10^{2}$	+	$1.84\times 10^{1}$	$5.60\times 10^{2}$	+	$8.80\times 10^{2}$	$1.20\times 10^{3}$	+	$1.16\times 10^{4}$	$5.16\times 10^{2}$	+	$2.54\times 10^{2}$	$5.70\times 10^{2}$	+	$8.25\times 10^{2}$	$5.21\times 10^{2}$	$5.87\times 10^{1}$
28	$3.96\times 10^{2}$	−	$1.78\times 10^{2}$	$3.53\times 10^{2}$	≈	$3.57\times 10^{3}$	$4.27\times 10^{2}$	+	$1.02\times 10^{3}$	$3.68\times 10^{2}$	−	$2.83\times 10^{3}$	$3.21\times 10^{2}$	−	$2.27\times 10^{3}$	$3.12\times 10^{2}$	$4.38\times 10^{2}$
29	$6.19\times 10^{2}$	+	$5.31\times 10^{3}$	$1.04\times 10^{3}$	+	$5.62\times 10^{4}$	$6.84\times 10^{2}$	+	$5.04\times 10^{3}$	$4.99\times 10^{2}$	−	$3.83\times 10^{3}$	$8.79\times 10^{2}$	+	$2.84\times 10^{4}$	$6.56\times 10^{2}$	$3.77\times 10^{3}$
30	$1.57\times 10^{4}$	+	$1.69\times 10^{7}$	$1.13\times 10^{5}$	+	$4.01\times 10^{9}$	$3.43\times 10^{5}$	≈	$3.32\times 10^{10}$	$2.23\times 10^{3}$	−	$5.05\times 10^{4}$	$8.94\times 10^{4}$	+	$5.65\times 10^{9}$	$2.86\times 10^{5}$	$1.28\times 10^{10}$
AR.21-30	**2.40**		2.80	4.90		5.00	3.90		3.80	2.90		3.10	4.40		4.40	2.50	**1.90**
AR.1-30	3.54		3.25	4.96		5.00	3.32		3.46	**2.64**		2.75	3.89		4.21	**2.64**	**2.32**
sum		13/3/14			21/3/6			10/5/15			8/6/16			16/3/11			

Note: The bold mark indicates that they are the best results among the algorithms.

**Table 2:** Comparison of statistical results evaluated by five algorithms on 50D CEC 2017 benchmark functions.

F	ABC			dynFWA			LOTFWA			PVADE			SPSO2011			QDF-TE	
	**MeanErr**	**Win**	**StdErr**	**MeanErr**	**Win**	**StdErr**	**MeanErr**	**Win**	**StdErr**	**MeanErr**	**Win**	**StdErr**	**MeanErr**	**Win**	**StdErr**	**MeanErr**	**StdErr**
1	$2.35\times 10^{3}$	+	$3.27\times 10^{6}$	$9.36\times 10^{3}$	+	$8.23\times 10^{7}$	$7.41\times 10^{2}$	−	$1.36\times 10^{6}$	$2.43\times 10^{3}$	+	$7.24\times 10^{6}$	$2.70\times 10^{3}$	+	$1.38\times 10^{7}$	$9.62\times 10^{2}$	$9.86\times 10^{5}$
2	$9.95\times 10^{21}$	+	$2.75\times 10^{45}$	$1.22\times 10^{5}$	−	$2.74\times 10^{11}$	$2.17\times 10^{24}$	+	$1.04\times 10^{50}$	$1.14\times 10^{15}$	+	$3.01\times 10^{31}$	$8.87\times 10^{-5}$	−	$1.34\times 10^{-9}$	$1.65\times 10^{11}$	$1.12\times 10^{23}$
3	$2.15\times 10^{5}$	+	$1.85\times 10^{8}$	$2.25\times 10^{-8}$	−	$1.25\times 10^{-15}$	$7.67\times 10^{3}$	+	$6.65\times 10^{6}$	$1.41\times 10^{1}$	−	$2.55\times 10^{2}$	$2.63\times 10^{-3}$	−	$1.76\times 10^{-4}$	$1.82\times 10^{1}$	$2.00\times 10^{2}$
AR.1-3	4.67		4.67	3.00		3.00	4.00		4.33	3.67		4.00	**2.67**		2.67	3.00	**2.33**
4	$3.05\times 10^{1}$	−	$9.52\times 10^{1}$	$1.18\times 10^{2}$	≈	$2.58\times 10^{3}$	$1.02\times 10^{2}$	≈	$6.97\times 10^{2}$	$1.10\times 10^{2}$	≈	$2.53\times 10^{3}$	$1.21\times 10^{2}$	+	$3.44\times 10^{3}$	$1.01\times 10^{2}$	$2.12\times 10^{3}$
5	$2.05\times 10^{2}$	+	$3.33\times 10^{2}$	$2.83\times 10^{2}$	+	$3.81\times 10^{3}$	$1.57\times 10^{2}$	+	$5.92\times 10^{2}$	$2.10\times 10^{2}$	+	$1.88\times 10^{3}$	$1.40\times 10^{2}$	+	$5.40\times 10^{2}$	$6.96\times 10^{1}$	$1.38\times 10^{2}$
6	0.00	−	0.00	$4.11\times 10^{1}$	+	$7.57\times 10^{1}$	$9.30\times 10^{-1}$	−	1.34	1.05	−	$9.72\times 10^{-1}$	$1.80\times 10^{1}$	+	$2.66\times 10^{1}$	2.57	$8.44\times 10^{-1}$
7	$2.32\times 10^{2}$	+	$3.71\times 10^{2}$	$4.52\times 10^{2}$	+	$1.05\times 10^{4}$	$1.24\times 10^{2}$	+	$2.52\times 10^{2}$	$2.83\times 10^{2}$	+	$3.56\times 10^{2}$	$2.25\times 10^{2}$	+	$1.98\times 10^{3}$	$1.16\times 10^{2}$	$1.37\times 10^{2}$
8	$2.12\times 10^{2}$	+	$3.98\times 10^{2}$	$2.83\times 10^{2}$	+	$6.31\times 10^{3}$	$1.76\times 10^{2}$	+	$8.72\times 10^{2}$	$2.08\times 10^{2}$	+	$1.43\times 10^{3}$	$1.47\times 10^{2}$	+	$4.53\times 10^{2}$	$7.01\times 10^{1}$	$1.33\times 10^{2}$
9	$8.61\times 10^{3}$	+	$4.21\times 10^{6}$	$1.10\times 10^{4}$	+	$1.05\times 10^{7}$	$2.53\times 10^{3}$	+	$2.33\times 10^{6}$	$4.04\times 10^{1}$	−	$2.33\times 10^{3}$	$1.45\times 10^{3}$	+	$7.09\times 10^{5}$	$3.33\times 10^{2}$	$2.56\times 10^{4}$
10	$2.56\times 10^{4}$	+	$1.27\times 10^{5}$	$6.45\times 10^{3}$	+	$7.18\times 10^{5}$	$4.67\times 10^{3}$	≈	$2.31\times 10^{5}$	$7.79\times 10^{3}$	+	$2.51\times 10^{6}$	$7.39\times 10^{3}$	+	$1.87\times 10^{6}$	$4.59\times 10^{3}$	$2.06\times 10^{5}$
AR.4-10	3.29		2.29	5.57		5.57	2.71		3.29	4.00		3.86	3.71		4.29	**1.71**	**1.71**
11	$2.49\times 10^{3}$	+	$1.24\times 10^{6}$	$2.13\times 10^{2}$	+	$3.45\times 10^{3}$	$3.72\times 10^{2}$	+	$6.36\times 10^{3}$	$1.54\times 10^{2}$	+	$1.55\times 10^{3}$	$1.78\times 10^{2}$	+	$1.51\times 10^{3}$	$1.33\times 10^{2}$	$4.80\times 10^{2}$
12	$4.87\times 10^{6}$	+	$2.90\times 10^{12}$	$5.01\times 10^{6}$	+	$7.93\times 10^{12}$	$6.95\times 10^{6}$	+	$1.03\times 10^{13}$	$1.38\times 10^{4}$	−	$7.94\times 10^{7}$	$2.19\times 10^{6}$	≈	$1.24\times 10^{12}$	$2.20\times 10^{6}$	$6.59\times 10^{11}$
13	$9.25\times 10^{3}$	−	$3.98\times 10^{7}$	$1.01\times 10^{5}$	+	$2.74\times 10^{9}$	$4.77\times 10^{4}$	+	$3.28\times 10^{8}$	$1.91E\times 10^{3}$	−	$7.90\times 10^{6}$	$7.20\times 10^{4}$	+	$2.02\times 10^{9}$	$3.28\times 10^{4}$	$6.92\times 10^{7}$
14	$1.16\times 10^{6}$	+	$2.49\times 10^{11}$	$2.98\times 10^{4}$	+	$7.09\times 10^{8}$	$1.97\times 10^{4}$	+	$2.19\times 10^{8}$	$2.04\times 10^{2}$	−	$2.09\times 10^{3}$	$8.68\times 10^{3}$	≈	$6.24\times 10^{7}$	$7.80\times 10^{3}$	$2.86\times 10^{7}$
15	$1.59\times 10^{4}$	+	$1.31\times 10^{7}$	$1.35\times 10^{4}$	≈	$8.13\times 10^{7}$	$1.77\times 10^{4}$	+	$6.08\times 10^{7}$	$3.73\times 10^{2}$	−	$1.34\times 10^{4}$	$2.80\times 10^{4}$	+	$2.91\times 10^{8}$	$1.11\times 10^{4}$	$1.93\times 10^{7}$
16	$1.25\times 10^{3}$	+	$4.25\times 10^{4}$	$1.66\times 10^{3}$	+	$1.81\times 10^{5}$	$1.25\times 10^{3}$	+	$5.29\times 10^{4}$	$6.77\times 10^{3}$	≈	$5.24\times 10^{4}$	$1.25\times 10^{3}$	+	$1.44\times 10^{5}$	$6.46\times 10^{2}$	$3.43\times 10^{4}$
17	$9.83\times 10^{2}$	+	$1.84\times 10^{4}$	$1.50\times 10^{3}$	+	$1.20\times 10^{5}$	$5.52\times 10^{2}$	≈	$2.10\times 10^{4}$	$6.16\times 10^{2}$	+	$2.87\times 10^{4}$	$1.11\times 10^{3}$	+	$5.25\times 10^{4}$	$5.34\times 10^{2}$	$2.54\times 10^{4}$
18	$1.90\times 10^{6}$	+	$5.14\times 10^{11}$	$1.90\times 10^{5}$	+	$5.34\times 10^{9}$	$2.32\times 10^{5}$	+	$1.11\times 10^{10}$	$5.47\times 10^{2}$	−	$1.98\times 10^{5}$	$6.84\times 10^{4}$	≈	$5.49\times 10^{8}$	$7.38\times 10^{4}$	$5.79\times 10^{8}$
19	$2.16\times 10^{4}$	−	$3.97\times 10^{7}$	$1.74\times 10^{4}$	−	$2.15\times 10^{8}$	$1.72\times 10^{5}$	≈	$1.59\times 10^{10}$	$1.18\times 10^{2}$	−	$1.46\times 10^{3}$	$6.04\times 10^{4}$	−	$2.89\times 10^{8}$	$1.20\times 10^{5}$	$1.99\times 10^{9}$
20	$8.16\times 10^{2}$	+	$2.21\times 10^{4}$	$1.20\times 10^{3}$	+	$1.13\times 10^{5}$	$5.72\times 10^{2}$	+	$1.92\times 10^{4}$	$3.01\times 10^{2}$	≈	$1.75\times 10^{4}$	$7.28\times 10^{2}$	+	$4.41\times 10^{4}$	$2.44\times 10^{2}$	$1.71\times 10^{4}$
AR.11-20	4.30		3.50	4.70		5.00	4.40		4.30	**1.50**		**1.80**	3.90		4.00	2.20	2.40
21	$3.91\times 10^{2}$	+	$5.07\times 10^{3}$	$4.74\times 10^{2}$	+	$3.63\times 10^{3}$	$1.50\times 10^{2}$	−	$1.72\times 10^{-16}$	$3.81\times 10^{2}$	+	$2.82\times 10^{3}$	$3.30\times 10^{2}$	+	$7.65\times 10^{2}$	$2.57\times 10^{2}$	$7.90\times 10^{1}$
22	$4.36\times 10^{3}$	−	$5.02\times 10^{6}$	$6.88\times 10^{3}$	+	$8.75\times 10^{5}$	$1.50\times 10^{2}$	−	$5.22\times 10^{-21}$	$1.02\times 10^{2}$	−	7.35	$6.12\times 10^{3}$	+	$9.03\times 10^{6}$	$4.51\times 10^{3}$	$3.22\times 10^{6}$
23	$6.59\times 10^{2}$	+	$1.61\times 10^{3}$	$7.66\times 10^{2}$	+	$8.30\times 10^{3}$	$1.29\times 10^{3}$	+	$2.85\times 10^{4}$	$5.55\times 10^{2}$	+	$3.87\times 10^{3}$	$5.99\times 10^{2}$	+	$2.22\times 10^{3}$	$5.04\times 10^{2}$	$2.26\times 10^{2}$
24	$9.64\times 10^{2}$	+	$2.46\times 10^{4}$	$8.52\times 10^{2}$	+	$9.89\times 10^{3}$	$3.26\times 10^{2}$	−	$2.25\times 10^{4}$	$6.20\times 10^{2}$	+	$3.78\times 10^{3}$	$6.62\times 10^{2}$	+	$1.03\times 10^{3}$	$5.61\times 10^{2}$	$1.86\times 10^{2}$
25	$5.02\times 10^{2}$	≈	$5.04\times 10^{2}$	$5.13\times 10^{2}$	≈	$1.55\times 10^{3}$	$5.00\times 10^{2}$	≈	$1.13\times 10^{3}$	$5.60\times 10^{2}$	+	$1.69\times 10^{3}$	$5.67\times 10^{2}$	+	$1.47\times 10^{3}$	$5.08\times 10^{2}$	$1.19\times 10^{3}$
26	$1.34\times 10^{3}$	−	$1.51\times 10^{6}$	$4.51\times 10^{3}$	+	$6.44\times 10^{5}$	$2.12\times 10^{2}$	−	$1.06\times 10^{3}$	$3.31\times 10^{2}$	−	$6.57\times 10^{3}$	$3.73\times 10^{3}$	+	$1.47\times 10^{6}$	$2.05\times 10^{3}$	$4.30\times 10^{4}$
27	$6.50\times 10^{2}$	≈	$6.19\times 10^{2}$	$8.98\times 10^{2}$	+	$1.62\times 10^{4}$	$2.46\times 10^{3}$	+	$6.62\times 10^{4}$	$6.98\times 10^{2}$	+	$7.18\times 10^{3}$	$9.56\times 10^{2}$	+	$1.33\times 10^{4}$	$6.50\times 10^{2}$	$7.99\times 10^{2}$
28	$4.73\times 10^{2}$	≈	$8.16\times 10^{1}$	$4.92\times 10^{2}$	≈	$7.58\times 10^{2}$	$5.00\times 10^{2}$	+	$2.11\times 10^{3}$	$5.13\times 10^{2}$	+	$1.12\times 10^{3}$	$5.02\times 10^{2}$	+	$5.18\times 10^{2}$	$4.84\times 10^{2}$	$4.83\times 10^{2}$
29	$1.07\times 10^{3}$	≈	$1.54\times 10^{4}$	$1.91\times 10^{3}$	+	$1.53\times 10^{5}$	$1.52\times 10^{3}$	+	$4.17\times 10^{4}$	$8.36\times 10^{2}$	−	$3.79\times 10^{4}$	$1.79\times 10^{3}$	+	$1.42\times 10^{5}$	$1.04\times 10^{3}$	$2.34\times 10^{4}$
30	$8.52\times 10^{5}$	−	$5.86\times 10^{9}$	$1.18\times 10^{7}$	−	$6.50\times 10^{12}$	$3.36\times 10^{6}$	−	$2.60\times 10^{12}$	$7.06\times 10^{5}$	−	$1.77\times 10^{10}$	$5.46\times 10^{6}$	−	$6.45\times 10^{11}$	$1.54\times 10^{7}$	$3.55\times 10^{12}$
AR.21-30	3.00		3.00	5.00		4.70	2.90		3.60	**2.80**		3.40	4.50		3.80	**2.80**	**2.50**
AR.1-30	3.75		3.32	4.79		4.75	3.46		3.82	2.86		3.07	3.89		3.86	**2.25**	**2.18**
sum		19/4/7			22/4/4			18/5/7			14/3/13			23/3/4			

Note: The bold mark indicates that they are the best results among the algorithms.

## Data Availability

Data are contained within the article.
